# Cognition and Cerebrovascular Reactivity in Midlife Women With History of Preeclampsia and Placental Evidence of Maternal Vascular Malperfusion

**DOI:** 10.3389/fnagi.2021.637574

**Published:** 2021-05-04

**Authors:** C. Elizabeth Shaaban, Caterina Rosano, Ann D. Cohen, Theodore Huppert, Meryl A. Butters, James Hengenius, W. Tony Parks, Janet M. Catov

**Affiliations:** ^1^Department of Epidemiology, University of Pittsburgh, Pittsburgh, PA, United States; ^2^Center for the Neural Basis of Cognition, University of Pittsburgh, Pittsburgh, PA, United States; ^3^Department of Psychiatry, University of Pittsburgh, Pittsburgh, PA, United States; ^4^Department of Radiology, University of Pittsburgh, Pittsburgh, PA, United States; ^5^Department of Bioengineering, University of Pittsburgh, Pittsburgh, PA, United States; ^6^Department of Pathology and Laboratory Medicine, Mount Sinai Hospital, Toronto, ON, Canada; ^7^Department of Laboratory Medicine and Pathobiology, University of Toronto, Toronto, ON, Canada; ^8^Department of Obstetrics, Gynecology and Reproductive Sciences, University of Pittsburgh, Pittsburgh, PA, United States

**Keywords:** arterial spin labeling, cerebral blood flow, cognitive function, hypercapnia, hypertensive disorders of pregnancy, vascular contributions to cognitive impairment and dementia

## Abstract

**Background:** Preeclampsia is emerging as a sex-specific risk factor for cerebral small vessel disease (SVD) and dementia, but the reason is unknown. We assessed the relationship of maternal vascular malperfusion (MVM), a marker of placental SVD, with cognition and cerebral SVD in women with and without preeclampsia. We hypothesized women with both preeclampsia and MVM would perform worst on information processing speed and executive function.

**Methods:** Women (*n* = 45; mean 10.5 years post-delivery; mean age: 41 years; 42.2% Black) were classified as preeclampsia-/MVM-, preeclampsia+/MVM-, or preeclampsia+/MVM+. Information processing speed, executive function, and memory were assessed. In a pilot sub-study of cerebrovascular reactivity (CVR; *n* = 22), cerebral blood flow during room-air breathing and breath-hold induced hypercapnia were obtained via arterial spin labeling MRI. Non-parametric tests and regression models were used to test associations.

**Results:** Between-group cognitive differences were significant for information processing speed (*p* = 0.02); preeclampsia+/MVM+ had the lowest scores. Cerebral blood flow increased from room-air to breath-hold, globally and in all regions in the three groups, except the preeclampsia+/MVM+ parietal region (*p* = 0.12). Lower parietal CVR (less change from room-air breathing to breath-holding) was correlated with poorer information processing speed (partial ρ = 0.63, *p* = 0.005) and executive function (ρ = 0.50, *p* = 0.03) independent of preeclampsia/MVM status.

**Conclusion:** Compared to women without preeclampsia and MVM, midlife women with both preeclampsia and MVM have worse information processing speed and may have blunted parietal CVR, an area important for information processing speed and executive function. MVM in women with preeclampsia is a promising sex-specific indicator of cerebrovascular integrity in midlife.

## Introduction

Preeclampsia, characterized by *de novo* hypertension and end-organ dysfunction (American College of Obstetricians and Gynecologists, 2020), occurs in as many as 8% of pregnancies and affects up to 15% of women (Auger et al., 2016; Garovic et al., 2020). A common finding in preeclampsia is insufficient vascular remodeling to develop the maternal side of the placenta, the maternal decidua, with lower perfusion and small vessel disease (SVD) in the placenta (Parks, 2015).

Preeclampsia is emerging as a sex-specific risk factor for vascular contributions to cognitive impairment and dementia (VCID) in later life (Theilen et al., 2016; Basit et al., 2018; Elharram et al., 2018; Gannon et al., 2019; Andolf et al., 2020). Studies indicate preeclampsia-related differences in information processing speed, executive function, and, to some extent, also memory (Brussé et al., 2008; Fields et al., 2017; Miller et al., 2019). Neuroimaging studies indicate preeclampsia-related gray matter atrophy (Mielke et al., 2016; Siepmann et al., 2017; Miller et al., 2019) and white matter hyperintensities (Wiegman et al., 2014; Siepmann et al., 2017). Recent evidence also suggests preeclampsia-related lower cerebrovascular reactivity (CVR), an early sign of cerebral SVD, using carotid or total brain transcranial Doppler 35–40 years after pregnancy (mean age, 60 years) (Barnes et al., 2018).

The reason for the occurrence of neurovascular and cognitive deficits in preeclampsia is unknown. Associations are overall independent of cardiometabolic risk factors and cardiovascular diseases. A possible explanation is that women with preeclampsia have more severe SVD not only in the placenta, but also affecting the cerebral vasculature, which can manifest as cerebral SVD later in life. Histopathological measures of placental SVD may hold the key to capturing SVD severity during pregnancy. Decidual vasculopathy and concomitant hypoxia/reperfusion lesions in the placenta are collectively termed maternal vascular malperfusion (MVM) (Parks, 2015). MVM is emerging as a risk factor for maternal vascular risk later in life for both large (Staff et al., 2010, 2013; Stevens et al., 2014, 2015; Catov et al., 2018; Holzman et al., 2020) and small vessels (Pepine et al., 2015; Bairey Merz, 2019). We have recently found that 8–10 years after pregnancy, women with MVM have excess masked hypertension (Catov et al., 2019), sublingual microvascular dysfunction (Hauspurg et al., 2020), and reduced coronary flow reserve (Countouris et al., 2020). However, prior studies of cerebrovascular integrity did not have direct measures of SVD severity during pregnancy, and hence could not assess the relationship between preeclampsia, MVM, and cerebral dysfunction. Our primary objectives were to test the associations of MVM and preeclampsia with cognition and to carry out a pilot study to assess the feasibility of measuring CVR in whole brain and regions of interest (ROIs) in these women. We hypothesized that women with both preeclampsia and MVM would perform worst on information processing speed and executive function. Our secondary aim was to explore associations of CVR with MVM, preeclampsia, and cognition.

## Methods

### Participants

**FIGURE 1:**
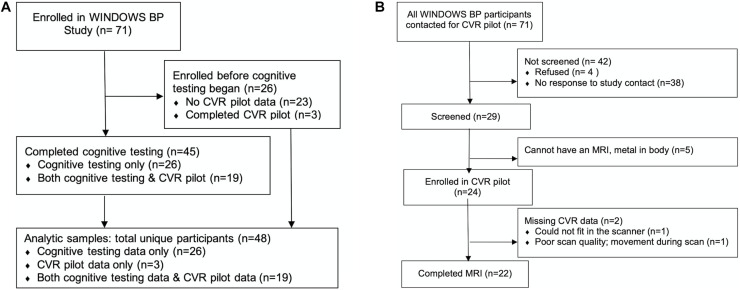
Study participation flow diagrams. CVR, cerebrovascular reactivity. **(A)** Overall study flow diagram and **(B)** detailed study flow diagram for the cerebrovascular reactivity (CVR) pilot study.

Participants were drawn from an observational study of women’s post-partum cardiovascular health, the WINDOWS BP Study (*N* = 71). Twenty-six women were enrolled before cognitive assessments began, and therefore *N* = 45 women were included in this study of cognitive assessments (see study participation flow in Figure 1A). We report neuroimaging pilot study results in *N* = 22. A detailed description of the neuroimaging study participation flow is provided in the Results section “Pilot Study Feasibility” and Figure 1B. WINDOWS BP participants were drawn from the parent WINDOWS Study (*N* = 498), an ancillary study to our pregnancy registry with data for all births from 2008 to 2010 at Magee-Womens Hospital, Pittsburgh, PA, United States (*N* = 6632); they were women who delivered a liveborn, singleton infant at Magee-Womens Hospital and who had a clinical placenta pathology report (for the index delivery). Extensive characterization of preeclampsia and placental MVM data were collected at the time of birth (2008–2010) and validated via chart review and blinded review of pathology specimens. Data on vascular and cardiometabolic factors were collected 8–10 years after delivery at the WINDOWS study visit, unless otherwise indicated. WINDOWS study participants were not currently pregnant or pregnant within the last 6 months. At the cognitive assessment study visit [mean (SD): 10.5 (0.6) years after delivery], cognitive testing and MRI were collected. Eligibility criteria for our MRI pilot study included that participants had no MRI contraindications such as claustrophobia or non-MRI cleared metal in the body. All WINDOWS study participants were stroke-free.

By design, we enrolled women with preeclampsia regardless of MVM status and women without preeclampsia had no MVM. We focused on placental severity as opposed to clinical preeclampsia severity, and we hypothesized that women with both preeclampsia and MVM would have the most adverse profile.

This study was approved by the University of Pittsburgh Institutional Review Board, and all participants provided written informed consent before any study procedures were carried out.

### MRI Pilot Study

In preparation for a larger planned study, we invited all participants of the WINDOWS BP study to complete an MRI with measures of CVR. We aimed to assess the feasibility of recruitment and carrying out our CVR imaging protocol and to obtain estimates of: CVR by study group, variance, and association effect sizes for cognition and CVR.

We follow the reporting guidance of Strengthening the Reporting of Observational Studies in Epidemiology (STROBE) (von Elm et al., 2007) and the Consolidated Standards of Reporting Trials (CONSORT) extension to randomized pilot and feasibility trials (Eldridge et al., 2016) based on recommendations for reporting non-randomized pilot studies that serve as preparation for larger studies and which test preliminary associations (Lancaster and Thabane, 2019).

### Predictors

#### Preeclampsia

Preeclampsia is based on the registry medical record data for the index delivery and validated via chart abstraction. Cases were identified based on American College of Obstetricians and Gynecologists (ACOG) criteria: new-onset hypertension (≥140/90 mmHg) after 20-weeks’ gestation accompanied by proteinuria or other organ involvement (American College of Obstetricians and Gynecologists, 2020).

To characterize the clinical severity of preeclampsia, we report the proportion of women with the following based on the index delivery: preeclampsia with severe features based on ICD-9 code, pulmonary edema, seizures, any headache, a pre-term delivery (<37 weeks’ gestation), and an infant that was small for gestational age. We also report gestational age and birth weight.

#### Maternal Vascular Malperfusion

Placental histopathology was used to detect MVM (Staff et al., 2010) using uniform consensus criteria (Khong et al., 2016). We classified MVM as present in the placenta if any of the following consensus rated features were identified: decidual vasculopathy, villous infarction, accelerated villous maturation, perivillous fibrin deposition, or intervillous fibrin deposition (defined in Supplementary Table 1). Specimens were retrieved and reviewed by an expert perinatal pathologist (WTP), blinded to all clinical information except gestational age. There was substantial agreement between the blinded review and the clinical report of MVM (kappa = 0.78). Cases with disagreement underwent an additional round of review for adjudication.

### Outcomes

#### Cognition

Cognitive assessments were administered by trained raters overseen by a neuropsychologist. Our domains of interest were information processing speed, executive function, and memory. The information processing speed domain included Digit Symbol Coding (Wechsler, 1997a) and Stroop Word Reading and Color Naming (Golden, 1978). Tests in the executive function domain included the Similarities and Matrix Reasoning subtests of the Wechsler Adult Intelligence Scale-III (Wechsler, 1997a) and Stroop Color Word Interference (Golden, 1978). Tests in the memory domain included Logical Memory Immediate and Delayed Recall (Wechsler, 1997b). Cognitive test z-scores were created, and domain composite z-scores were then calculated as the average of the test *z*-scores for that domain. A higher score is better for all domains.

#### Cerebrovascular Reactivity

Cerebrovascular reactivity, a measure of vessel response to a vasoactive stimulus (e.g., increased CO_2_), is an excellent marker of cerebral SVD (Bakker et al., 1999; Rane et al., 2018). We evaluated global and regional CVR non-invasively as change in blood flow from room-air breathing to blood flow during a 24-s breath-hold using a validated arterial spin labeling MRI imaging protocol (Urback et al., 2017; Cohen and Wang, 2019). We used this less invasive approach to induce hypercapnia for our pilot study in order to assess feasibility of, lay groundwork for, and justify using the more invasive and logistically complicated approach of CO_2_ challenge in a larger study (e.g., administration of CO_2_ requires an Investigational New Drug approval). We did not measure end-tidal CO_2_, but breath-holding calibrates well against direct administration of CO_2_ to induce hypercapnia (Kastrup et al., 2001; Bulte et al., 2009), and this breath-holding approach has been used to successfully induce hypercapnia in other population-based studies (Haight et al., 2015). Details of our standardized CVR protocol and methods can be found in the Supplementary Methods section and details of ROIs in Supplementary Table 2.

### Covariates and Other Study Characteristics

Systolic blood pressure (SBP) and diastolic blood pressure (DBP) were measured in a standardized way by trained study staff based on our established protocol using a Microlife A6 PC / BP 3GUI-8X (Guangdong, China). Following a 5-min rest, BP was measured three times on the non-dominant arm with 1-min intervals with an appropriate cuff size based on arm measurement. The average of these three measures was used for data analyses. Body mass index (BMI) at the parent study visit was calculated as $\frac{w\,e\,i\,g\,h\,t\,\left(k\,g\right)}{{\left[h\,e\,i\,g\,h\,t\,\left(m\right)\right]}^{2}}$ based on measured height (using a stadiometer) and weight (Tanita scale TBF-300A) after women removed their shoes, socks, and excessive clothing. Waist circumference was measured with a tape measure at end-expiration at the level of the iliac crest. Total cholesterol, high-density lipoprotein cholesterol (HDL-C), low-density lipoprotein cholesterol (LDL-C), triglycerides, glycated hemoglobin (HbA1c), and high sensitivity C-reactive protein (hs-CRP) were measured in fasting blood samples at the University of Pittsburgh Medical Center (UPMC) Presbyterian Hospital Automated Testing Laboratory. Any values that were less than the assay’s level of detection were set to the test value/2. Smoking status (yes/no) was recorded from the registry medical record during pregnancy. Gestational diabetes (yes/no) was any pregnancy prior to the parent study visit with gestational diabetes based on the registry medical record. Menopausal status (yes/no) was assessed via reproductive history questionnaire, aligned with the landmark SWAN study to record date of last menstruation, hysterectomy, oophorectomy history, and use of hormonal contraception (Tseng et al., 2012; Cortes et al., 2017).

### Statistical Analysis

We calculated sample characteristics overall and by preeclampsia/MVM status in the 45 cognitive assessment study participants. They are presented as means and standard deviations for continuous variables or numbers and percentages for categorical variables. Group comparisons were carried out using ANOVA for normally distributed continuous variables, Kruskal–Wallis tests for non-normally distributed continuous variables, and Fisher’s exact tests for categorical variables. We did not conduct inferential statistical tests on the preeclampsia severity variables due to small sample and cell sizes.

For primary aim hypothesis testing involving cognition, we set alpha to 0.05 and corrected for multiple comparisons using the Bonferroni correction. Because the neuroimaging aim of this study was a pilot study, we wanted to increase the likelihood of exploring promising results in our larger planned study, making us more concerned with erroneously ruling out a potential finding (false negative) than with false positives (Rothman, 1990). Thus, we set alpha at 0.10, and we did not correct for multiple comparisons in analyses involving CVR. Statistical analyses were carried out in SPSS version 25.0, SAS version 9.4, and *R* version 4.0.2.

#### Cognition

Forty-five women had cognitive assessments (Figure 1). Differences in information processing speed, executive function, and memory by preeclampsia/MVM status were tested using ANOVA. Significant tests were repeated adjusting for education alone and in combination with SBP, BMI, or both SBP and BMI using generalized linear models (GLMs). For any cognitive domain significantly associated with preeclampsia/MVM status in education adjusted models, we followed up with Bonferroni adjusted pairwise comparisons, and we also tested whether cognitive performance differences were driven by preeclampsia or MVM using ANOVA and education adjusted GLMs. For all of these models, education was treated as a continuous variable.

#### Pilot Study Feasibility

We report descriptive statistics as *N* (%) for participants’ response to invitation to participate, screening, enrollment, and usable CVR data.

#### CVR

Twenty-two participants had usable CVR data (Figure 1). We analyzed CVR (change from room-air breathing to breath-hold) and also computed percent signal change as (mean of breath-hold − mean of room-air breathing) / mean of room-air breathing × 100; higher CVR values indicate increased flow in response to breath-holding. Due to small sample size, we tested within preeclampsia/MVM group change from room-air breathing to breath-hold using Wilcoxon Signed Rank tests (non-parametric paired tests). We also tested between group differences in room-air breathing, breath-hold, and percent change using Kruskal–Wallis tests.

#### Associations of CVR With Cognition

Nineteen participants had data on both CVR and cognition (Figure 1). We evaluated associations of global and regional CVR with cognition independent of preeclampsia/MVM status using Spearman partial correlations. We further adjusted these partial correlations one at a time for years of education, gestational diabetes, menopause, preeclamspia severity, pre-term birth, and any variables which differed significantly by preeclampsia/MVM status.

## Results

Participant characteristics are presented in Table 1. Overall, the participants were about 31 years of age at delivery and 41 years of age at the time of cognitive assessments. About 42% of the participants were of Black race/ethnicity, and most had greater than high school education. Among the participants, *n* = 24 had neither preeclampsia nor MVM, *n* = 8 had preeclampsia only, and *n* = 13 had preeclampsia with MVM. There were no significant differences in participant characteristics by preeclampsia/MVM status with the exception of DBP. Women with both preeclampsia and MVM had the highest DBP (*p* = 0.04, Table 1).

**TABLE 1:** Characteristics of study participants overall and by preeclampsia and maternal vascular malperfusion status.

**Characteristic**	**Overall (*n* = 45)**		**PreE-/MVM- (*n* = 24)**		**PreE+/MVM-(*n* = 8)**		**PreE+/MVM+ (*n* = 13)**		***p***
Age at delivery, years	30.6	5.6	31.3	5.3	27.9	6.2	30.8	5.6	0.31
Age at Cognitive test, years	41.0	5.5	41.6	5.3	38.5	5.9	41.5	5.5	0.35
Education, years*	15.2	2.6	15.4	2.6	15.3	2.8	14.8	2.5	0.77
Education, <HS	10	22.2%	5	20.8%	1	12.5%	4	30.8%	0.70
Smoking during pregnancy^#^	7	16.3%	5	20.8%	0	0%	2	18.2%	0.46
Gestational diabetes	6	13.3%	3	12.5%	2	25%	1	7.7%	0.60
Black	19	42.2%	11	45.8%	3	37.5%	5	38.4%	0.49
BMI, kg/m^2^ at parent study visit	31.6	7.7	29.9	7.6	31.6	7.9	34.5	7.4	0.22
Waist circumference, inches	39.7	7.1	38.3	7.2	40.2	9.0	42.0	5.4	0.33
SBP, mmHg*	119.7	14.4	117.2	15.6	118.3	11.4	125.0	13.4	0.15
DBP, mmHg*	78.4	10.1	75.6	9.8	78.0	10.1	83.8	9.0	0.04
Total cholesterol, mg/dl^∧^	181.0	41.8	182.8	36.3	162.7	43.6	187.5	50.4	0.44
LDL-C, mg/dl^∧^	100.9	33.2	99.5	29.4	87.0	35.0	111.0	38.1	0.30
HDL-C, mg/dl^∧^	58.9	16.4	62.6	17.2	58.0	19.5	52.5	11.7	0.20
Triglycerides, mg/dl^∧^*	106.7	67.3	104.6	65.7	88.3	35.7	120.5	83.1	0.80
hsCRP, mg/dl^∧^*	0.4	0.9	0.3	0.4	1.1	2.0	0.3	0.3	0.60
HbA1c, percent^∧^*	5.8	1.8	5.4	0.5	6.4	3.1	6.2	2.4	0.28
Post-menopausal	3	6.7%	2	8.3%	0	0%	1	7.7%	>0.99
Severe preeclampsia	–	–	–	–	1	12.5%	6	46.2%	–
Pulmonary edema	–	–	–	–	0^∧∧^	0%	0^$^	0%	–
Seizures	–	–	–	–	0^∧∧^	0%	0^$^	0%	–
Headache^&^	–	–	–	–	1	12.5%	3^@^	23.1%	–
Pre-term birth	–	–	–	–	2	25.0%	4	30.8%	–
Gestational age, weeks	–	–	–	–	37.0	2.0	36.3	3.8	–
Small for gestational age	–	–	–	–	1	12.5%	5	38.5%	–
Birth weight	–	–	–	–	3,137.4	629.8	2,637.4	1,080.9	–

^#^*N* = 43; ^∧^*N* = 44; ^∧∧^*N* = 7; ^&^*N* = 6; ^$^*N* = 10; and ^@^*N* = 11. Mean and standard deviations or numbers (%) are presented for continuous and categorical variables, respectively. *P*-values for continuous variables are from ANOVA for normally distributed variables and from Kruskal–Wallis tests for non-normally distributed variables (indicated by *), and *p*-values for categorical variables are from Fisher’s exact tests. BMI, body mass index; DBP, diastolic blood pressure; HbA1c, glycated hemoglobin; HDL-C, high-density lipoprotein cholesterol; HS, high school; hsCRP, high sensitivity c-reactive protein; LDL-C, low-density lipoprotein cholesterol; MVM, maternal vascular malperfusion; PreE, preeclampsia; SBP, systolic blood pressure.

Clinical features of preeclampsia severity by MVM status are reported in Table 1. Compared to women without MVM, women with MVM were likelier to have severe preeclampsia, report headaches, and to have an infant that was small for gestational age, although numbers were small, so we did not perform statistical tests of these differences. No women had pulmonary edema or seizures.

### Cognition

**FIGURE 2:**
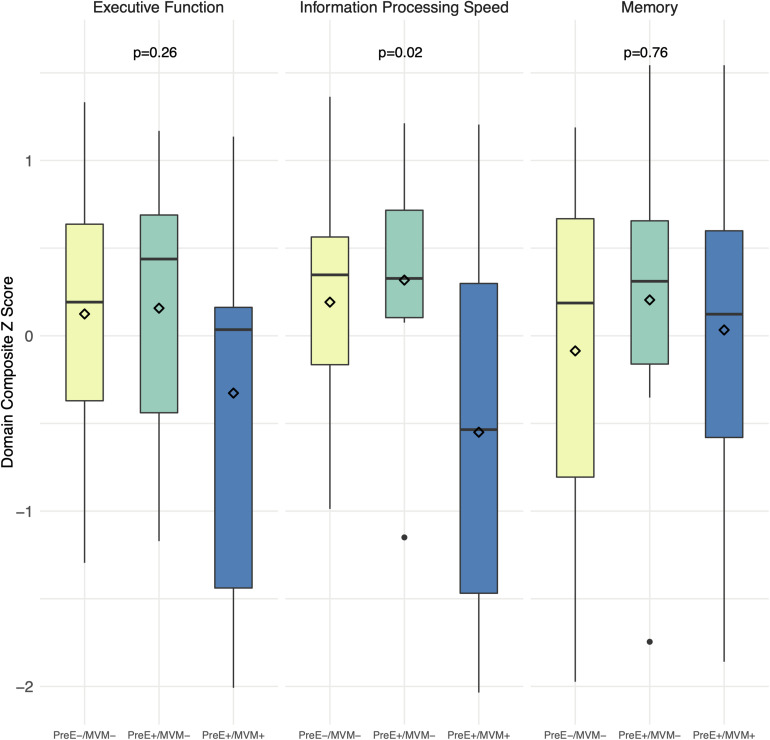
Cognitive performance by preeclampsia and maternal vascular malperfusion status. Boxplots of unadjusted composite *z*-scores for each cognitive domain. Domain composite *z*-scores are average *z*-scores of the tests comprising the domain. Diamonds = mean score. *P* values are from ANOVAs. MVM, maternal vascular malperfusion; PreE, preeclampsia.

Boxplots of unadjusted cognitive scores by preeclampsia/MVM status are shown in Figure 2. We found women with both preeclampsia and MVM had the worst information processing speed, mean (SD): preeclampsia-/MVM-, +0.19 (0.63); preeclampsia+/MVM-, +0.32 (0.72); preeclampsia+/MVM+, −0.55 (1.06) (overall *p* = 0.02). In pairwise comparisons, preeclampsia-/MVM- vs. preeclampsia+/MVM+ were significantly different at a Bonferroni adjusted alpha of 0.017 for three comparisons (pairwise *p* = 0.01), but not preeclampsia+/MVM- vs. preeclampsia+/MVM+ (*p* = 0.06) or preeclampsia-/MVM- vs. preeclampsia+/MVM- (*p* = 0.64). Preeclampsia+/MVM+ women also had the lowest executive function scores, although the overall difference was not significant (overall *p* = 0.26). There was no significant difference in memory scores (overall *p* = 0.76). Differences in information processing speed appeared driven more by MVM rather than preeclampsia (MVM+ vs. MVM-, *p* = 0.004; preeclampsia+ vs. preeclampsia-, *p* = 0.11). Overall associations of preeclampsia/MVM status with information processing speed remained similar after adjustment for years of education, SBP, and BMI.

### Pilot Study Feasibility

Detailed pilot study participation flow is shown in Figure 1B. Out of the 71 WINDOWS BP participants who were invited to participate in the CVR pilot study, 38 (53.5%) were non-responsive. The frank refusal rate was 5.6% (*n* = 4). We screened 29 (40.8%) for participation, and of those, 5 (17.2%) were MRI screen failures. We enrolled 24 (33.8%) participants. Two participants were missing CVR data: one participant was unable to fit in the scanner, and one had poor scan quality due to motion while in the scanner. This resulted in 22 (31.0%) participants with usable CVR data.

### Cerebrovascular Reactivity

Across the full study sample, cerebral blood flow increased during breath-hold compared to room-air breathing (average global CVR increase: 12%). This was true of global blood flow within the three preeclampsia/MVM groups as well. But using regional spatial distribution, preeclampsia+/MVM+ women demonstrated a less robust CVR response in the parietal lobe; they showed no statistically significant difference between room-air breathing and breath-hold, while preeclampsia-/MVM- and preeclampsia+/MVM- women did (Table 2). All groups significantly increased blood flow from room air breathing to breath-hold in the other ROIs. Although there was a consistent pattern across ROIs for preeclampsia+/MVM+ women to have the lowest cerebral blood flow under room-air breathing conditions, all between-group differences for cerebral blood flow during room-air breathing or breath-hold, or for percent signal change—CVR— were not statistically significant (all *p* > 0.1).

**TABLE 2:** Cerebral blood flow during room-air breathing and breath-hold stratified by preeclampsia and maternal vascular malperfusion status.

	**PreE-/MVM- (*n* = 9)**			**PreE+/MVM- (*n* = 5)**			**PreE+/MVM+ (*n* = 8)**		
	**Room- air**	**Breath- hold**	**Within arm p**	**Room- air**	**Breath- hold**	**Within arm p**	**Room- air**	**Breath- hold**	**Within arm p**
Global	11.7 (1.6)	12.9 (2.2)	0.038	12.0 (1.4)	13.6 (0.8)	0.080	11.1 (2.0)	12.4 (1.8)	0.012
Parietal	10.8 (2.1)	11.6 (2.5)	0.051	11.0 (1.2)	12.5 (1.1)	0.080	10.1 (1.9)	10.9 (1.7)	0.123
Precuneus	14.2 (1.9)	15.3 (2.3)	0.038	14.2 (1.4)	15.9 (0.6)	0.080	12.8 (1.9)	14.6 (3.4)	0.012
Anterior Cingulate	14.3 (2.5)	16.1 (3.1)	0.028	15.1 (1.8)	17.4 (2.2)	0.043	13.2 (2.6)	15.6 (3.5)	0.012
Posterior Cingulate	15.7 (2.0)	17.0 (3.1)	0.066	16.1 (2.5)	17.7 (1.5)	0.080	14.2 (1.9)	16.9 (3.5)	0.012

**N* = 22. Values are mean (standard deviation). *P*-values are based on Wilcoxon Signed Rank tests, non-parametric paired tests. MVM, maternal vascular malperfusion; PreE, preeclampsia.*

### Associations of CVR With Cognition

Scatterplots, partial correlations, and *p*-values of cognitive scores by CVR for significant correlations are shown in Supplementary Figure 1. We found significant correlations of lower CVR in the parietal lobe with worse information processing speed and executive function (Supplementary Figure 1), but not memory (*p* = 0.16), independent of preeclampsia/MVM status. Anterior and posterior cingulate CVR were significantly inversely associated with memory (Supplementary Figure 1), but not information processing speed or executive function (all *p* > 0.1). Lower global CVR was significantly correlated with worse information processing speed (Supplementary Figure 1), but not executive function or memory (*p* > 0.1), independent of preeclampsia/MVM status. These relationships survived additional one at a time adjustments for education, DBP, gestational diabetes, menopause, preeclampsia severity, and pre-term birth, except: parietal CVR with executive function adjusted for education (ρ = 0.38, *p* = 0.13) and both anterior cingulate CVR with memory (ρ = −0.39, *p* = 0.11) and global CVR with information processing speed (ρ = 0.41, *p* = 0.11) adjusted for preeclampsia severity. Correlations of CVR in other ROIs with cognition were not significant (all *p* > 0.1).

## Discussion

Consistent with our hypothesis, midlife women with a history of both preeclampsia and MVM had poorer information processing speed compared to women with neither preeclampsia nor MVM, and nearly so compared to women with preeclampsia only; these differences appeared driven by MVM rather than preeclampsia. While preeclampsia-related poorer information processing speed has been previously reported (Brussé et al., 2008; Fields et al., 2017; Miller et al., 2019), our results that women with both MVM and preeclampsia had worse cognitive function are novel. Our pilot study demonstrated the feasibility of enrolling such women originally drawn from a birth registry for our future larger study incorporating advanced neuroimaging, with 41% of women in our parent study screened, 34% enrolled, and 31% with usable neuroimaging data. Further characterizing the cerebrovascular integrity of women with histories of preeclampsia and MVM in a larger study is important, as our pilot results demonstrate that the women with both preeclampsia and MVM also appeared to have blunted CVR localized in the parietal lobe, a region important for information processing speed and executive function. Our results raise the intriguing possibility that small vessel impairments in the placenta may additionally mark the subset of women with preeclampsia who are susceptible to poorer cognition and cerebral SVD. Future studies to identify mechanisms are needed.

The possible blunted CVR response in women with both preeclampsia and MVM may be localized in the parietal lobe for several reasons. Both in older and younger adults with high vascular risk, cerebral SVD is more common in fronto-parietal and subcortical areas, due to poor collateral vascularity (watershed areas) (Pantoni, 2010). Of note, in this group, the parietal lobe may have been more susceptible to lower CVR because it had lower baseline flow. Cerebral SVD in fronto-parietal areas affect executive function regulation (Wardlaw et al., 2013), planning, problem-solving, and decision-making (Salthouse, 1996; Erel and Levy, 2016; Igelström and Graziano, 2017; Ardila et al., 2018; Bubb et al., 2018; Froudist-Walsh et al., 2018; Gratton et al., 2018; Nguyen et al., 2019; Worringer et al., 2019). In older age, cerebral SVD is an established risk factor for dementia (Saxton et al., 2004; Backman et al., 2005; Rapp and Reischies, 2005; Rosano and Newman, 2006; Debette et al., 2019), and associations of lower CVR with dementia risk have been documented (Di Marco et al., 2015; De Silva and Faraci, 2016; Nelson et al., 2016; Peng et al., 2018). While direct evidence for these associations is sparser in midlife (Jorgensen et al., 2018), lower neurocognitive integrity in midlife may reduce brain reserve, increasing vulnerability to age-related risk factors, and lowering the threshold for clinical manifestations of brain changes (Wang et al., 2017; Windham et al., 2019; Xu et al., 2019). Our work extends this to indicate that neurocognitive differences in the reproductive years may be detectable, a critical step to identification of high-risk individuals who may be susceptible to later life impairments. In sum, focal changes in CVR in early midlife women with preeclampsia may be an early risk factor for development of VCID.

### Strengths and Limitations

The strengths of our study include our evaluation of women in early midlife at a time in which risk factors for VCID are more amenable to intervention. Our study sample included a large proportion of Black women. Prior work has primarily been in white women, yet hypertensive disorders of pregnancy and pregnancy-related morbidity and mortality are disproportionately experienced by Black women (Breathett et al., 2014; Shahul et al., 2015; Miller et al., 2020). We also applied rigorous measurement of study variables. Our characterization of preeclampsia was drawn from our birth registry, with the medical record reviewed and diagnosis adjudicated and applied according to current American College of Obstetricians and Gynecologists criteria. We evaluated MVM, a novel measure of SVD in the placenta, a vascular bed not typically considered in cerebral SVD studies. Our evaluation of MVM was based on expert review of placental histopathology, with chance of rater bias reduced by blinding to nearly all participant clinical information, including preeclampsia diagnosis. We used a functional measure of cerebral microvascular dysfunction—CVR—which is capable of capturing early changes in cerebrovascular integrity; these changes can appear without yet corresponding to the structural degradation of tissue-based markers of cerebral SVD (Shaaban et al., 2019). We used a standardized breath-hold-based CVR protocol to enhance consistency across participants. In addition, our imaging methods captured two novel aspects of CVR compared to prior methods relying on transcranial Doppler: the spatial distribution of cerebral blood flow at the regional level, and the contribution of signal from smaller sized vessels. Transcranial doppler is limited to capturing changes in large arteries and precludes parsing vascular changes regionally as we have done (Blair et al., 2016). Our regional approach allowed us to connect cognitive performance in specific domains with cerebral SVD in brain regions that subserve these functions.

There are several limitations which should be kept in mind when considering our study results. We did not have additional details on severity and neurological symptoms of preeclampsia beyond what we reported in Table 1. Our neuroimaging sample was small, and the associated analyses were, by design for a pilot study, exploratory. We noted large standard deviations in our CVR measures; thus absence of significant relationships may be true or may be due simply to small sample size and large variability. We also used breath-holding to induce hypercapnia and did not measure end-tidal CO_2_. These pilot study results lay groundwork for our larger planned study where we will be able to further evaluate and confirm these results using CO_2_ challenge to induce hypercapnia; this is expected to elicit a stronger response with a larger effect size compared to breath-holding, making it easier to detect. This study provides only one timepoint of CVR and cognitive measures; change over time was not evaluated, and we also cannot know what these participants’ cognitive performance and CVR were prior to our assessments nor prior to preeclampsia or MVM onset. Understanding how these factors in midlife evolve and relate to late-life cognitive function will require longitudinal studies.

### Future Directions

Our results suggest that regional CVR may more precisely reveal preeclampsia-related injury than global CVR, especially in relatively young women. Therefore, regional CVR measures should be incorporated in future longitudinal studies linking preeclampsia to long-term brain health. Future studies can incorporate other methods that induce a greater hypercapnic response, such as CO_2_ challenge. Larger, longitudinal studies using a broader-based cognitive battery are warranted to further evaluate the relationships of preeclampsia, MVM, cognition, and CVR. Specifically, a better understanding of how preeclampsia and MVM impact information processing speed and whether cognitive functions worsen from early midlife into late-life will identify if pregnancy is a key window of opportunity for risk identification and targeted intervention.

## Data Availability Statement

Data are available from the University of Pittsburgh Institutional Data Access/Ethics Committee for researchers who meet the criteria for access to confidential data. Requests to access the datasets should be directed to JC, jmcst43@pitt.edu.

## Ethics Statement

The studies involving human participants were reviewed and approved by University of Pittsburgh Institutional Review Board. The patients/participants provided their written informed consent to participate in this study.

## Author Contributions

JC, CR, WP, AC, TH, and CS contributed to conception and design of the study. CR and CS performed the statistical analysis. JC, CR, WP, MB, AC, TH, and CS interpreted the data for the work. CS wrote the first draft of the manuscript. JC and CR drafted sections of the manuscript. All authors contributed to manuscript revision and read and approved the submitted version.

## Conflict of Interest

The authors declare that the research was conducted in the absence of any commercial or financial relationships that could be construed as a potential conflict of interest.
