# Supplementary material for: Cognition and Cerebrovascular Reactivity in Midlife Women With History of Preeclampsia and Placental Evidence of Maternal Vascular Malperfusion
Source: Front Aging Neurosci. 2021 May 4;13:637574. doi: 10.3389/fnagi.2021.637574 (PMC8129174; doi:10.3389/fnagi.2021.637574)
Supplement: Supplementary file 1 [file Table_1.docx]

**Supplementary Methods**

**Cerebrovascular reactivity (CVR)**

Flow estimates were averaged across four breath-hold sequences, which were structured as follows: room-air breathing for 48 seconds, post-expiration breath-hold for 24 seconds, resume room-air breathing. The breath-holding procedure was explained to the participants and they engaged in a practice round with the research associate before entering the scanner, followed by another practice round if they had any difficulty with the first practice round. Specifically, they were advised to take a breath in and then breathe out all of their air during a countdown from 5 to 1; hold their breath for 24 seconds; at the end of another countdown from 5 to 1, they could breathe normally. The participants were guided through this task while in the scanner via E-Prime directions that were displayed on screen. Participants wore a chest strap to verify they were not breathing during breath-hold. If they did breathe during a round of breath-hold, the research associate re-instructed them over the intercom during the room-air breathing round before the next breath-hold round.

We measured global (whole brain) CVR and regional CVR in the following regions of interest (ROIs) generated according to the Desikan-Killiany atlas using Freesurfer (<https://surfer.nmr.mgh.harvard.edu/>)(Desikan et al., 2006): parietal, precuneus, and anterior and posterior cingulate (Desikan-Killiany regions comprising study ROIs are listed in Supplementary Table 2). ROIs were selected based on neuroanatomy underpinning information processing speed and executive function, areas which would be adversely impacted by poor vascular function due to non-overlapping vascularization (watershed areas), and regions used in our prior studies of Alzheimer’s disease pathology in cognitively normal older adults (Cohen et al., 2013).

Imaging was carried out at the University of Pittsburgh MR Research Center using a Siemens 3-Tesla scanner. Women were not fasting, and they were not instructed to alter typical hydration, caffeine, or medication use prior to scanning. We collected a T1 Magnetization-Prepared Rapid Gradient Echo (MPRAGE) structural image (0.8mm isotropic (192 x 300 x 320); TR=2400ms, TE=2.24ms, FA=8 degrees) (Greve and Fischl, 2009). We used a quantitative imaging of perfusion using a single subtraction, second version (QUIPSS-II) pulsed arterial spin labeling (ASL) sequence (Luh et al., 1999) with a 2D GRE spiral readout (4x4x10mm with 10mm slice gap (64x64x9); 91 TRs; TR=2400ms, TE=12ms, TI=800ms, FA=90 degrees). ASL was motion corrected using FSL-FLIRT(Woolrich et al., 2009) following boundary-based registration to the T1 structural image (Greve and Fischl, 2009). The interweaved label and control pulsed ASL volumes were separated using the FSL-BASIL program (Chappell et al., 2008). We used the registered Freesurfer volume parcellation to normalize the gray matter flow estimates to the white matter and to extract weighted-average ROI flow estimates. Due to this normalization, units are relative. We averaged the 24 seconds before each breath-hold period and the 24s during each breath-hold excluding the initial two TRs for each block to avoid transition effects and allowing a 24 second recovery period following each breath-hold.

| **Supplementary Table 1. Consensus diagnostic criteria for maternal vascular malperfusion in the placenta** | |
| --- | --- |
| Decidual vasculopathy | Incomplete, abnormal remodeling of maternal vessels supplying the placenta manifest as >1 of the following: 1) Absence of vascular remodeling (smooth muscle wall in >1 vessel). 2) Mural hypertrophy of decidual arterioles (thickening of the muscle wall of a vessel leaving a luminal diameter <30%). 3) Fibrinoid necrosis (degeneration of >1 vessel wall). 4) Atherosis (foamy macrophages within >1 vessel wall). |
| Villous infarction | Devitalization of a region of placental villi due to obstruction of the underlying maternal blood flow |
| Accelerated villous maturation | At least two specific pathologic changes in the villous architecture: 1) Advanced villous maturation (increase in the percentage of villi containing a syncytial knot); 2) Decrease in the percentage of intermediate villi and/or distal villous hypoplasia (zones of abnormally long, thin, unbranched terminal villi). |
| Perivillous fibrin deposition | Irregular zones of fibrinoid material tightly encasing the entrapped villi. |
| Intervillous fibrin deposition | Increased percentage (3% is the upper limit) of small foci of fibrinoid material within or adjacent to villi. |

**Supplementary Table 1.** Criteria are based on the Amsterdam placental workshop group consensus statement on the sampling and definitions of placental lesions.(Khong et al., 2016)

| **Supplementary Table 2. Atlas-based regions comprising cerebral blood flow regions of interest** | | | |
| --- | --- | --- | --- |
| Anterior Cingulate | | |  |
|  | Rostral Anterior Cingulate | |  |
|  | Caudal Anterior Cingulate | |  |
| Posterior Cingulate | | |  |
|  | Posterior Cingulate | |  |
|  | Isthmus Cingulate | |  |
| Parietal | |  |  |
|  | Inferior Parietal | |  |
|  | Superior Parietal | |  |
|  | Supramarginal | |  |
| Precuneus | | |  |
|  | Precuneus | |  |
| Global | |  |  |
|  | Rostral Anterior Cingulate | | Medial Orbito Frontal |
|  | Caudal Anterior Cingulate | | Insula |
|  | Accumbens |  | Superior Temporal |
|  | Caudate |  | Middle Temporal |
|  | Putamen |  | Banks of the Superior Temporal Sulcus |
|  | Rostral Middle Frontal | | Inferior Parietal |
|  | Superior Frontal | | Superior Parietal |
|  | Parstriangularis | | Supramarginal |
|  | Frontal Pole |  | Precuneus |
|  | Parsopercularis | | Posterior Cingulate |
|  | Caudal Middle Frontal | | Isthmus Cingulate |
|  | Lateral Orbito Frontal | |  |

**Supplementary Table 2.** Regions are based on the Desikan-Killiany atlas.

**Supplementary Figure 1. Spearman partial correlations of cerebrovascular reactivity and cognitive performance**


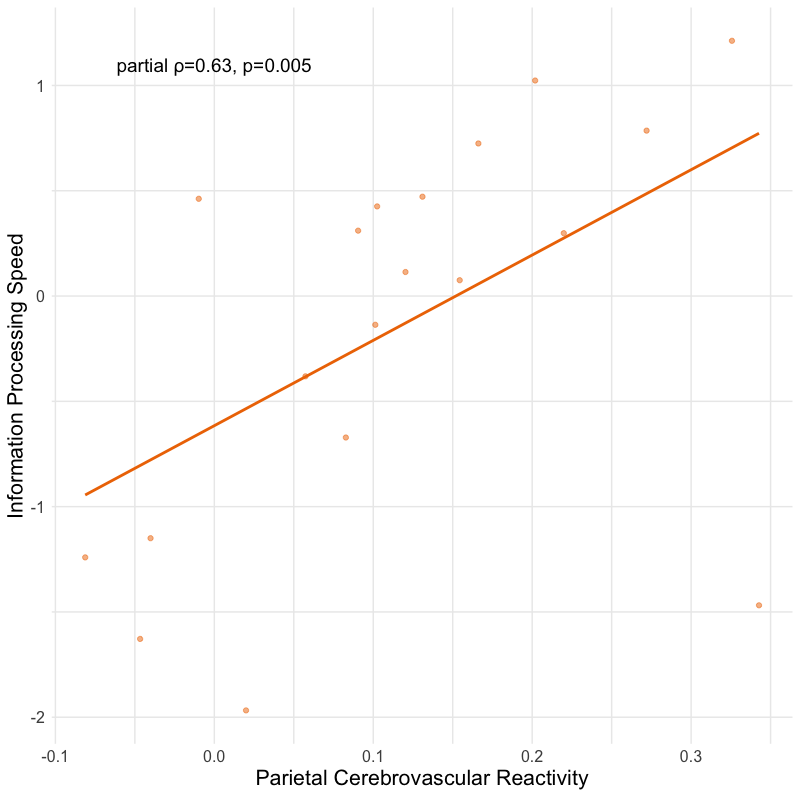

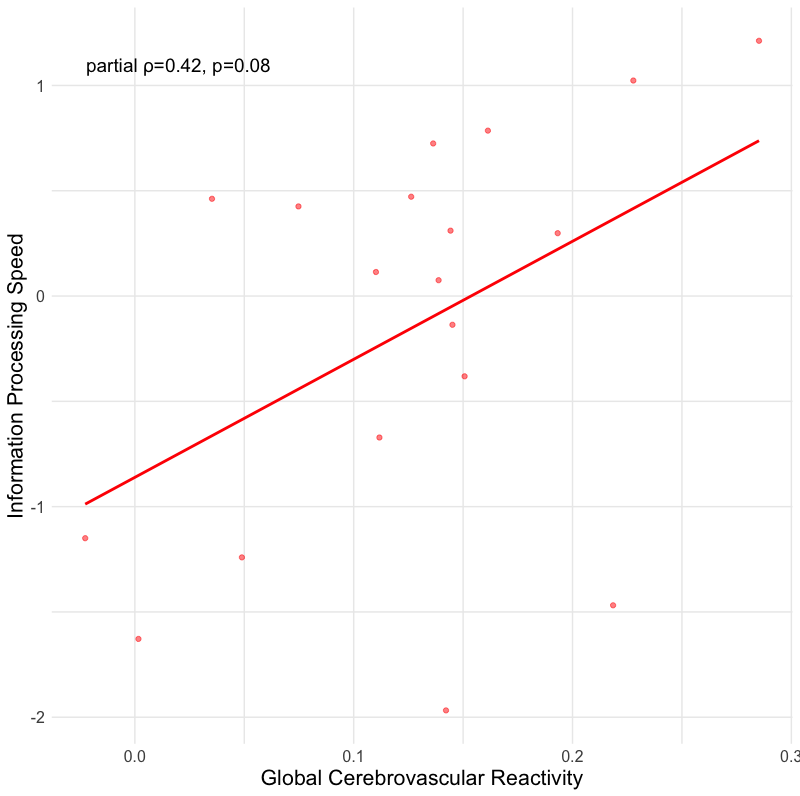


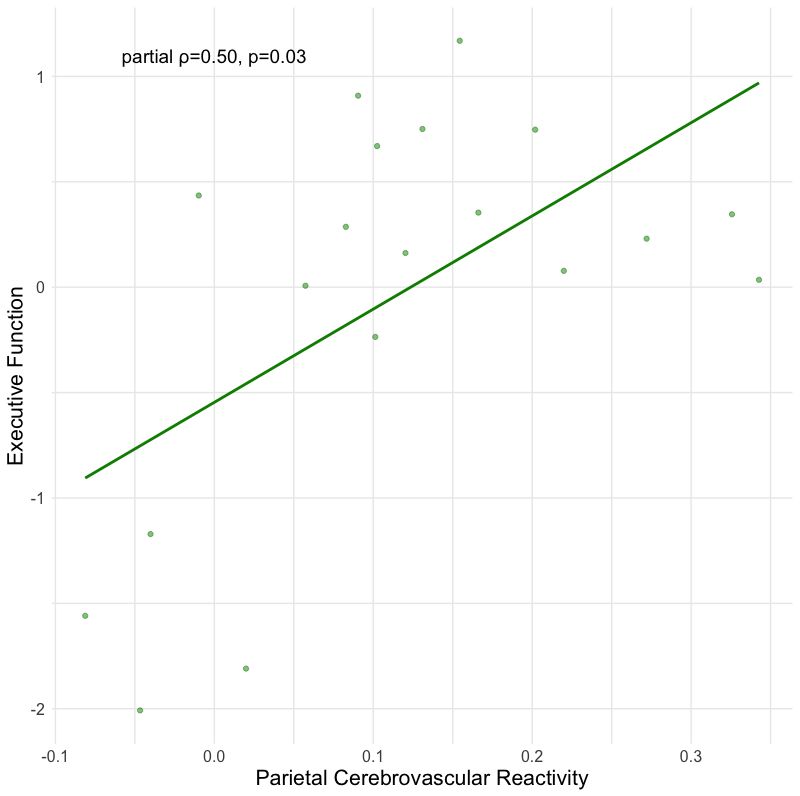


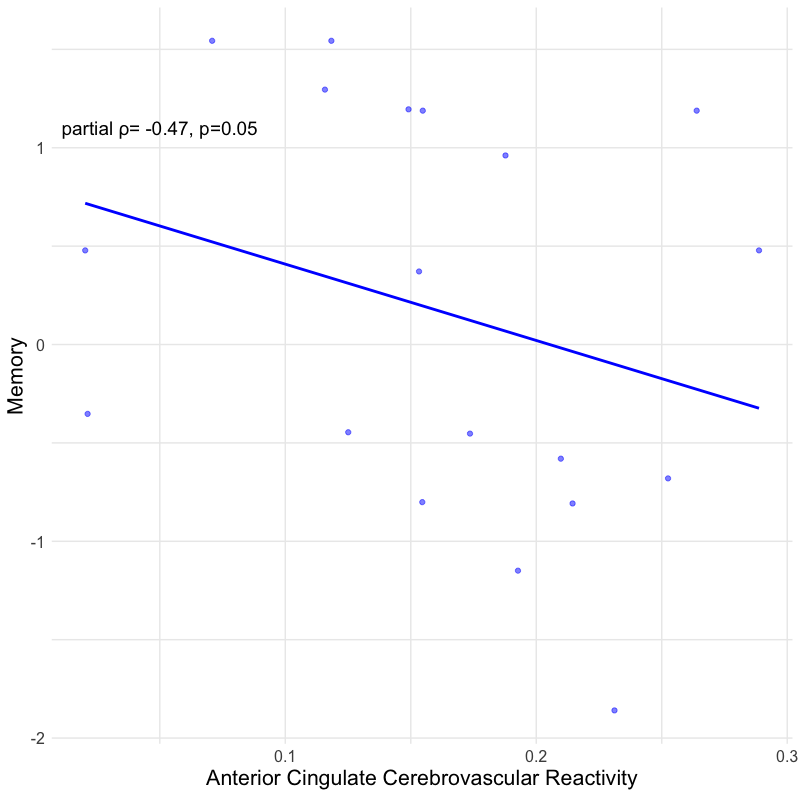

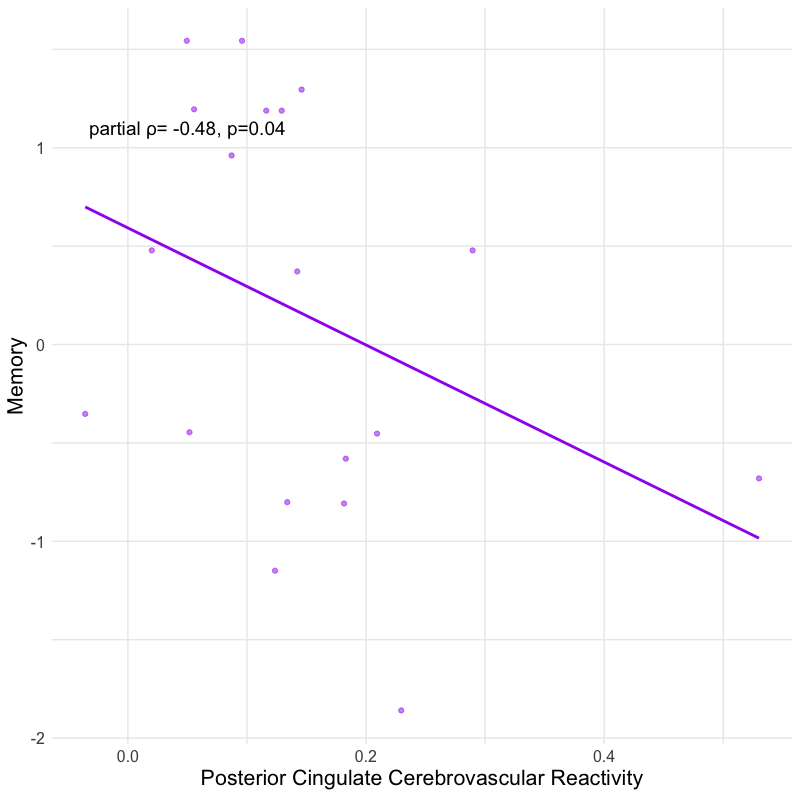


**Supplementary Figure 1.** Cognitive scores are domain composite z-scores. Cerebrovascular reactivity is proportional signal change; multiply by 100 to get percent signal change. Correlations partial out preeclampsia/maternal vascular malperfusion (MVM) status.

**REFERENCES**

Chappell, M.A., Groves, A.R., Whitcher, B., and Woolrich, M.W. (2008). Variational Bayesian inference for a nonlinear forward model. *IEEE Transactions on Signal Processing* 57(1)**,** 223-236.

Cohen, A.D., Mowrey, W., Weissfeld, L.A., Aizenstein, H.J., McDade, E., Mountz, J.M., et al. (2013). Classification of amyloid-positivity in controls: comparison of visual read and quantitative approaches. *Neuroimage* 71**,** 207-215. doi: 10.1016/j.neuroimage.2013.01.015.

Desikan, R.S., Segonne, F., Fischl, B., Quinn, B.T., Dickerson, B.C., Blacker, D., et al. (2006). An automated labeling system for subdividing the human cerebral cortex on MRI scans into gyral based regions of interest. *Neuroimage* 31(3)**,** 968-980. doi: 10.1016/j.neuroimage.2006.01.021.

Greve, D.N., and Fischl, B. (2009). Accurate and robust brain image alignment using boundary-based registration. *Neuroimage* 48(1)**,** 63-72. doi: 10.1016/j.neuroimage.2009.06.060.

Khong, T.Y., Mooney, E.E., Ariel, I., Balmus, N.C., Boyd, T.K., Brundler, M.A., et al. (2016). Sampling and Definitions of Placental Lesions: Amsterdam Placental Workshop Group Consensus Statement. *Arch Pathol Lab Med*. doi: 10.5858/arpa.2015-0225-CC.

Luh, W.M., Wong, E.C., Bandettini, P.A., and Hyde, J.S. (1999). QUIPSS II with thin-slice TI1 periodic saturation: a method for improving accuracy of quantitative perfusion imaging using pulsed arterial spin labeling. *Magn Reson Med* 41(6)**,** 1246-1254. doi: 10.1002/(sici)1522-2594(199906)41:6<1246::aid-mrm22>3.0.co;2-n.

Woolrich, M.W., Jbabdi, S., Patenaude, B., Chappell, M., Makni, S., Behrens, T., et al. (2009). Bayesian analysis of neuroimaging data in FSL. *Neuroimage* 45(1 Suppl)**,** S173-186. doi: 10.1016/j.neuroimage.2008.10.055.
